# Retrospective Clinical and Radiological Outcomes after Robotic Assisted Bicompartmental Knee Arthroplasty

**DOI:** 10.1155/2015/747309

**Published:** 2015-09-03

**Authors:** Cuneyt Tamam, Johannes F. Plate, Marco Augart, Gary G. Poehling, Riyaz H. Jinnah

**Affiliations:** Department of Orthopaedic Surgery, Wake Forest University School of Medicine, Winston Salem, NC 27157, USA

## Abstract

*Purpose*. Bicompartmental knee arthroplasty (BiKA) is a favorable alternative to total knee arthroplasty for degenerative disease limited to two knee compartments. Recently developed robotic-assisted systems improved the clinical efficacy of unicompartmental knee arthroplasty by providing enhanced component positioning with dynamic ligament balancing. The purpose of this study was to evaluate the short-term outcomes of patients, undergoing bicompartmental knee arthroplasty at a single institution by a single surgeon using a robotic-assisted system. It was hypothesized that robotic assisted BiKA is a prevailing choice for degenerative disease limited to two knee compartments with good functional results.* Methods*. A search of the institution's joint registry was conducted to identify patients that underwent robotic-assisted BiKA of the patellofemoral compartment and the medial or lateral compartment.* Results*. A total number of 29 patients (30 BiKA) with a mean age of 63.6 years were identified who received a patellofemoral resurfacing in combination with medial or lateral compartment resurfacing. Twenty-four out of 29 patients had good to excellent outcome.* Conclusion*. Robotic assisted bicompartmental arthroplasty using broad indications and only excluding patients with severe deformity and those that have less than 4 mm of joint space in the surviving compartment demonstrated 83% good to excellent results.

## 1. Introduction

Bicompartmental knee arthroplasty (BiKA) is a favorable alternative to total knee arthroplasty (TKA) for degenerative disease limited to two knee compartments. Most commonly, BiKA is a combination of medial and patellofemoral compartment resurfacing [1]. In contrast to total knee arthroplasty, bicompartmental knee arthroplasty preserves the uninvolved compartment and cruciate ligaments theoretically leading to advanced stability, improved proprioception, and more physiologic knee kinematics.

In the conventional unicompartmental knee arthroplasty (UKA) technique, the quadriceps tendon is split and the patella is dislocated and everted. Minimally invasive surgical (MIS) techniques have been introduced for unicompartmental knee arthroplasty to decrease surgical trauma, postoperative pain, and mobilization time, thus achieving a decreased hospitalization time, a quicker postoperative rehabilitation, and better functional outcomes [2, 3]. Even with MIS techniques, UKA systems offer limited joint visualization and potentially lead to malalignment. These systems are very dependent on surgeon's judgment and experience regarding prosthesis implantation. Recently published data suggest no advantages at a mid-term followup for the minimally invasive procedure in comparison to a conventional UKA [4].

Recently developed robotic-assisted systems improved the clinical efficacy of knee arthroplasty by providing enhanced component positioning with dynamic ligament balancing. Based on preoperative computed tomography (CT), constructed 3D model planning, and active surgeon-controlled cutting with robot-imposed limitations, these “semiactive” systems allow more accurate implant placement and may improve outcomes of BiKA.

The purpose of this study was to evaluate the short-term outcomes of patients, undergoing BiKA at a single institution by a single surgeon using a robotic-assisted system. Our hypothesis is that robotic assisted bicompartmental knee arthroplasty is a prevailing choice for degenerative disease limited to two knee compartments with good functional results.

## 2. Materials and Methods

A search of the institution's joint registry was conducted to identify patients that underwent robotic-assisted BiKA of the patellofemoral compartment and the medial or lateral compartment between December 2009 and April 2012. Approval was obtained by our institution's Institutional Review Board. A retrospective chart review of patient demographics, body mass index (BMI), American Society of Anesthesiologists (ASA) Class, operative data, the length of hospital stay, and complications was performed.

Body mass index is categorized as BMI < 25 kg/m^2^ normal, BMI between 25–29 kg/m^2^ overweight, 30–39 kg/m^2^ obese, and BMI > 40 kg/m^2^ as morbid obese [5].

The indications for BiKA were severe pain and difficulty with walking and performing daily activities with degenerative arthritis in one tibiofemoral compartment and the patellofemoral compartment, and there should be no pain, tenderness on palpation, or crepitus in the third compartment. The patient should have a good passive range of motion (at least 15°–100°). The ligaments should be functionally intact. The acceptable deformity should be correctible varus deformity up to 15°, valgus deformity up to 17°, flexion contracture up to 15°, and tibial shift maximum of 10 mm. BMI up to 74 kg/m^2^ and patellofemoral arthritis up to a Kellgren-Lawrence Grading Scale 4 were within the limits of indications of BiKA. Contraindications included joint instability, less than 4 mm of joint space of the surviving medial or lateral compartment, and inflammatory arthritis [6]. Upper age was no restriction.

The patients were evaluated preoperatively and postoperatively. After the operation the patients were followed up after 2 weeks, 6 months, and 1 year. After a year the patients were followed up annually. At each stage radiographs were repeated. Data collection was performed by a research nurse and measurements of radiographs were performed by 2 orthopedic surgeons.

Prior to operation, weight bearing radiographs including long-leg AP, AP, and flexion lateral and sunrise views were taken. Postoperatively AP and flexion lateral and sunrise views were assessed to monitor the lower extremity alignment and prosthesis alignment.

**Figure 1:**
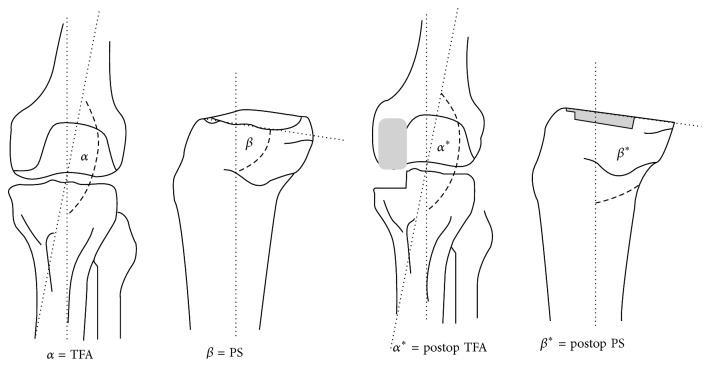
The femorotibial angle (FTA, *α*) formed by the intersection of the anatomical femoral and tibial axes. The tibial posterior slope angle (PS, *β*) was evaluated in preoperative lateral radiographs by considering the intersection of a line perpendicular to the articular surface of the medial tibial plateau and the anatomical mediolateral axis (i.e., the line between points situated at 10 and 20 cm from the plateau and located midway between the two cortices). In postoperative radiographs, it was determined as the angle formed by the intersection of the anatomical tibial axis and the horizontal axis of the tibial component [4].

**Figure 2:**
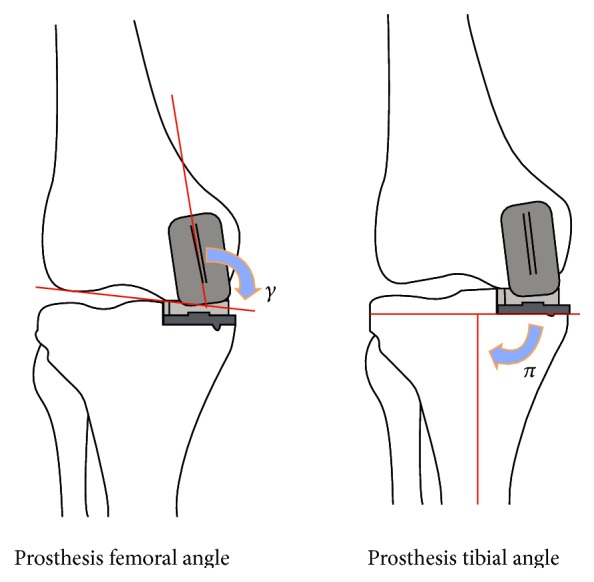
Prosthesis-femoral angle (*γ*) in the coronal plane formed by the intersection of the long axis of femoral prosthesis and the axis perpendicular to a line that passes by the distal femoral condyles and prosthesis-tibial angle (*π*) in the coronal plane formed by the intersection of the anatomical tibial axis and the line perpendicular to the tibial component [5].

Pre- and postoperative radiographs were compared and the femorotibial angle (FTA, *α*), the tibial posterior slope angle (PS, *β*), prosthesis-femoral angle (*γ*), and prosthesis-tibial angle (*π*) were evaluated (Figures 1 and 2).

The accuracy of implant positioning was determined using standard anteroposterior and lateral radiographs. The angles alpha, beta, and delta regarding the implant position, as illustrated in Figure 1, were radiologically assessed.

The optimal values for the angles were between 80° and 100° for angle (implant orientation femur AP) corresponding to a ±10° varus-valgus range, 83–92° for angle (tibial slope), and 80–95° for angle (implant orientation tibia AP), which corresponds to a range of 10° varus −5° valgus [7].

The clinical results assessed with the Oxford Knee Score (OKS) scored from 0 to 48. Scores >19 indicate poor results, 20–29 poor to fair, 30–39 fair to good, and 40–48 excellent outcome.

### 2.1. Statistical Methods

Continuous variables were compared with Student's *t*-test, paired sample *t*-test, or a Mann-Whitney *U* test when significant nonnormality was identified in the data. Each observer and the interobserver variation was analyzed using interobserver correlation coefficient. The Pearson correlation coefficient was used for correlation. It was accepted as statistically significant when the *p* value was < 0.05 with the confidence interval as 95%.

## 3. Preoperative Planning and Operative Technique

The patients scheduled to have robotically assisted arthroplasty underwent multislice computerized tomography of the operative knee. A three-dimensional reconstruction (3D) of the knee was created to be used for customized preoperative planning and registering.

Based on the preoperative 3D model, femoral and tibial implant position, coronal and sagittal alignment, overall leg alignment, gross anatomical deformities (cyst and vacuole), overlapping of the components in extension, geometric alignment of varus/valgus of femoral component to the varus of the tibia implant, and tibial implant positioning relative to the posterior tibial wall can be planned.

A mini-medial or lateral parapatellar or mini-midvastus arthrotomy is used as surgical approach depending on the surgeon's preference. The proximal extent of the incision is typically approximately 1–3 cm above the proximal pole of the patella and the distal extent just proximal to the tibial tubercle. Intraoperatively, navigation pins were used. Anatomic landmarks are used to register the patient to the robot following intracortical placement of the femoral and tibial marker array.

The knee is then ranged through a number of flexion-extension cycles. For a varus knee a valgus stress is then applied by the surgeon to open up the medial compartment and bring the knee into its “natural” alignment. The ligament balance is then analyzed and displayed by the computer system in real time as deviation from the optimal tracking pattern of the prosthesis calculated by the computer in millimeters (mm) during the flexion-extension cycle at 0°, 30°, 60°, 90°, and 120° of flexion. The system also calculates the flexion/extension gaps and graphically displays them. The prosthesis is then moved on the computer to normalize and correct the abnormalities found in the flexion/extension gaps.

The femoral condyle and tibial plateau are carved by a physician controlled but haptically guided high-speed burr on the robotic arm to accept the implants. The trochlear area is prepared in a similar fashion. The bone resection areas are defined by the system, and boundaries for the cutting instrument are set to prevent cutting into areas outside these boundaries.

The patellar articular surface is cut with an oscillating saw and guide apparatus. All patients received an unlinked modular bicompartmental prosthesis that included a UKA with an inlay all-polyethylene tibial component, onlay style femoral components, and an all-polyethylene dome shaped patellar component. BiKA and PFA utilized implants were Mako Restoris MCK (Mako Surgical Inc., Fort Lauderdale, FL). All implants were cemented.

## 4. Results

A total number of 29 patients (30 BiKA) with a mean age of 63.6 years (range 39 to 82) were identified who received a patellofemoral resurfacing in combination with medial (25, 83%) or lateral (5, 17%) compartment resurfacing. Fifty-seven percent of BiKAs were on the right knee. In this series, none of the patients had undergone prior distal tibial tubercle realignment procedures or periarticular osteotomies.

The mean BMI was 34.7 kg/m^2^ (SD 9.49, range 21.5 to 63.5) with 66% of bicompartmental surgeries being performed in patients with a BMI greater than 30 kg/m^2^. Eight patients were morbid obese and 11 were obese. 62% of the patients were female. 66% of patients had a preoperative ASA class of III and 30% had preoperative ASA class of II. The mean length of surgery was 40.5 minutes (range 23 to 151). The mean length of hospital stay was 39.82 ± 24.55 hours. One patient (3%, patellofemoral and medial compartment) underwent intraoperative lateral retinacular release.

At a mean followup of 27 months (range 12 to 54), no symptomatic venous thromboembolic complications occurred and no cases of patellar instability were observed. Two patients had superficial wound infection. Two patients (6%, 1 patellofemoral and lateral compartment, 1 patellofemoral and medial compartment) underwent arthroscopic debridement of loose cement fragments following BiKA. One patient (3%, patellofemoral and lateral compartment) required two arthroscopic debridements for a periprosthetic joint infection and loose body removal. No other knees required secondary surgery after BiKA. There were no component revisions or conversion to total knee arthroplasty noted during the follow-up period. The overall complication rate was 17.2%.

The mean oxford knee score was improved by  18 ± 6 points (range 10–28 points) to 36.43 ± 8.56 points (range 8–48 points) (*p* < 0.0001).

There was 1 patient with poor functional outcome. Twenty-four out of 29 patients (83%) had good to excellent outcome. There was no statistical correlation between OKS and the alignment angles.

The mean tibiofemoral angle was changed from degree 7 ± 5.48 of varus preoperatively to degree 3 ± 3.83 of valgus postoperatively. The mean correction angle was 2° (range −2° to 9.5°) (*p* = 0.019).

The mean tibial posterior slope was changed from degree 83.5 ± 1,38 preoperatively to degree 84.27 ± 1.56 postoperatively. The mean ProsFA and ProsTA was 91.05° ± 3.5 and 85.35° ± 3.35, respectively.

## 5. Discussion

Our study has found that with broad indications for bicompartmental knee arthroplasty we had an 83% good or excellent result with a minimum followup of 1 year (range 12 to 54). The outcomes of bicompartmental knee arthroplasty are related to a variety of factors, including patient-related factors, implant design, alignment, and fixation. Patient selection is a major concern that influences the outcomes in BiKA. In our study, we accepted broader inclusion criteria than generally accepted criteria defined by Kozinn and Scott [8]. The inclusion criteria required a range of motion of at least 90°, with correctible varus deformity up to 15°, valgus deformity up to 17°, flexion contracture up to 15°, tibial shift maximum of 10 mm, and no upper age restriction. The classic indications were osteoarthritis or osteonecrosis in either the medial or lateral compartment; age >60 years with a low demand for activity; weight <82 kg (181 lb); minimal pain at rest; range of motion (ROM) arc >90° with <5° flexion contracture; an angular deformity <15° that is passively correctable to neutral. Specific contraindications to UKA are a diagnosis of inflammatory arthritis; patient age <60 years; high patient activity level; pain at rest (which may indicate an inflammatory component to the arthropathy); patellofemoral pain or exposed bone in the patellofemoral joint or opposite compartment.

The ideal body mass index or cutoff weight for uni/bicompartmental knee arthroplasty patient still remains controversial. In our study, the mean BMI was 34.7 kg/m^2^. 66% of our bicompartmental surgeries were performed in patients with a BMI greater than 30 kg/m^2^. The functional outcome for good to excellent results was 83%. Berend et al. stated that a BMI greater than 32 kg/m^2^ was a predictor of failure and reduced survivorship [9]. Supporting this data, Heck et al. reported that the patients heavier than an arbitrary cutoff weight of 81 kg had significantly higher risk of failure and body weight more than 82 kg is an absolute contraindication [10].

Conversely, Naal et al. found no statistically significant difference in the early failure rates between patients who had BMIs below 25 kg/m^2^, between 25 and 29.9 kg/m^2^, or greater than 30 kg/m^2^ [11].

In another study, Tabor et al. found that obese patients had higher survival when compared with those who were not obese [12]. Kuipers et al. stated no early difference in revision rates between obese and nonobese patients [13]. The rate of lateral unicompartmental knee arthroplasty in our study accounts for 17% which is inconsistent with literature in which the rate is only 5–10% of all uni/bicompartmental arthroplasty cases [14, 15].

Bicompartmental knee arthroplasty has been done with two philosophically different femoral component designs, either with modular unlinked components which are what was done in this study or with a single monolithic design with linked between the patello- and tibiofemoral components [16].

The monolithic implant for BiKA has the challenge for the appropriate in sizing and implant alignment due to variability in coronal alignment and morphology of the distal femur [17]. Modular femoral implant for BiKA allows independent sizing and orientation of the individual components in each compartment.

In their study, Palumbo et al. concluded that the implantation of the monolithic bicompartmental prosthesis (Journey-Deuce) was an unreliable method to treat degenerated medial and PF compartments. They observed persistent knee pain and reduced function with a high incidence of conversion to TKA [18].

Morrison et al. had a revision rate of 14% of their 21 Journey-Deuce BiKA to TKA for persistent pain after 1 year postoperatively with a trend for increased revision rate at 2 years of followup [5]. For tibial components of UKAs, the first designs were cemented fixed-bearing all-polyethylene UKA [19]. Fixed-bearing tibial components have low conformity between the femoral and tibial components with low contact areas allowing for unconstrained movements between the femur and tibia controlled only by the ligamentous apparatus [20].

In 1986, Goodfellow and O'Connor described a mobile-bearing metal backed tibial component that allowed a more conformed surface than with a fixed-bearing UKA and therefore larger contact areas and lower contact stresses, which would theoretically improve wear characteristics [21].

In their* in vitro* study, Burton et al. concluded that both mobile- and fixed-bearing UKA devices when compared with clinically successful TKA devices achieved substantially reduced* in vitro* wear rates.

The study also demonstrated that wear was reduced with the fixed-bearing UKA compared with the mobile-bearing UKA [20]. Recent papers comparing clinical, radiological, and kinematic outcomes of fixed-to-mobile-bearing tibial components revealed similar improvements and outcomes [22–24].

The optimal positioning of the prosthesis in TKA is one of the most important determinants of a good clinical outcome and longevity of the device [25]. Correspondingly, in BiKA, there is no well-defined, precise range of coronal and sagittal optimal positioning for the components in UKA and BiKA [7, 26].

However, there is an agreement that variance beyond a safe range can predispose to complications. The angle tolerances specified for radiographic alignment of component implantation have been generally accepted as follows: between 80° and 100° corresponding to a range of ±10° varus/valgus for prosthesis-femur angle, 80–95° (10° varus to 5°) for prosthesis-tibia angle, and 83–92° for tibial slope angle [7, 26, 27].

In the current study the radiographic alignment of component implantation was within the safe range of general acceptance: 91.05 ± 3.5 for prosthesis-femur angle, 85.35 ± 3.35 for prosthesis-tibia angle, and 84.27 ± 1.56 for tibial slope angle (*p* = 0.016).

The wide range of the limits for the optimal positioning of the prosthesis components is a controversial question. Gulati stated that the primary reason for wide acceptable limits is that leg alignment depends on component thickness rather than alignment in UKA and thus BiKA [28]. However, Swienckowski and Page II reported that coronal malalignment of the tibial component beyond 3° was predisposed to failure [29].

The postoperative limb alignment is another important determinant of clinical outcome and longevity [30, 31]. However, no general agreement on correct postoperative limb alignment of BiKA has been achieved.

In our study, the mean tibiofemoral angle was changed from degree 7 ± 5.48 of varus preoperatively to degree 3 ± 3.83 of valgus postoperatively, with a mean correction angle of 2° (range −2° to 9.5°) (*p* = 0.019). The mean posterior tibial slope was changed from degree 83.5 ± 1.38 preoperatively to degree 84.27 ± 1.56 postoperatively.

Some authors stated that the aim of surgery is to simply replace a worn intra-articular joint surface and restore the mechanical axis of the lower limb to its physiological position before the onset of degenerative changes [32]. Other authors advocated restoring the mechanical axis to pass through the center of the knee joint [33, 34]. Fisher et al. recommended a concept of slight undercorrection for limb alignment. The recommended postoperative alignment for varus knees has a range from 0° to 5° of tibiofemoral valgus, with correction to 3° or 4° of tibiofemoral valgus. For lateral unicompartmental knee arthroplasty, 7° to 8° of tibiofemoral valgus has been recommended [35]. Gulati et al. considered 5° to 10° valgus to be the normal alignment [31].

Because of linear relationship between anterior tibial translation and posterior tibial slope, there is a higher risk of tibial component loosening in patients with posterior tibial slope higher than 7° [36].

The literature advocates relative undercorrection of the limb alignment with the presumption that overcorrection increases the risk of degeneration of the opposite tibiofemoral compartment [37]. Although debate remains about the amount of undercorrection ideal for an UKA, overcorrection beyond 0° has almost universally been discouraged [38]. There is also a controversy that undercorrection improves the outcome or that overcorrection causes progression. Gulati et al. revealed that varus malalignment does not compromise the functional outcome for UKA [31].

The complications seen in our study were 2 superficial wound infections, 2 postoperative loose fragments, and 1 periprosthetic joint infection and loose body, comprising an overall complication rate of 17.2%. Morrison et al. observed an overall complication rate of 28.6% [6].

In our study, bicompartmental arthroplasty, without distinction whether lateral or medial, reliably alleviated pain and improved the Oxford Knee Scores, from 18 ± 6 (range 10–28) to 36.43 ± 8.56 (range 8–48) (*p* < 0.0001). There was 1 patient with poor functional outcome. 24 out of 29 patients (83%) had good to excellent outcome. The Oxford scores were considered the most relevant and easiest function-related questionnaires. Our results are consistent with the literature. Liddle et al. matched the outcomes after total and unicompartmental knee arthroplasty in their study and found out that UKA resulted in significantly better early outcomes, in terms of both joint specific outcome and health-related quality of life. The OKS results of UKA were similar to our study [39].

There was no statistical correlation between OKS and the limb alignment angles. There was, also, no statistical correlation between OKS and BMI above and below 30.

Achieving reliable alignment of the components in BiKA using conventional approaches is difficult on a consistent basis. Computer navigation was introduced to improve the positioning based on the patients individual anatomy and increase the accuracy of UKA [40]. Navigation systems have been shown to reduce the number of alignment outliers that resulted from conventional instruments [41]. The development and introduction of minimally invasive surgical techniques had the potential to compromise implant alignment accuracy by decreasing the sight during operation [42, 43].

Robotic assistance refined and enhanced the improvements seen with computer navigation in increasing the accuracy of bone preparation techniques [44, 45]. Tactile robots are tools acting as virtual cutting guides or templates, permitting surgeons freely to remove bone within a preplanned cutting volume and prohibiting from removing bone outside that volume [46]. Cobb et al. first reported a prospective comparison of a tactile guided, robot-assisted UKA with conventional UKA. They demonstrated that a significant improvement in implant placement and accurate leg alignment can be achieved successfully with the aid of a semiactive robot system in UKA [44].

**Figure 3:**
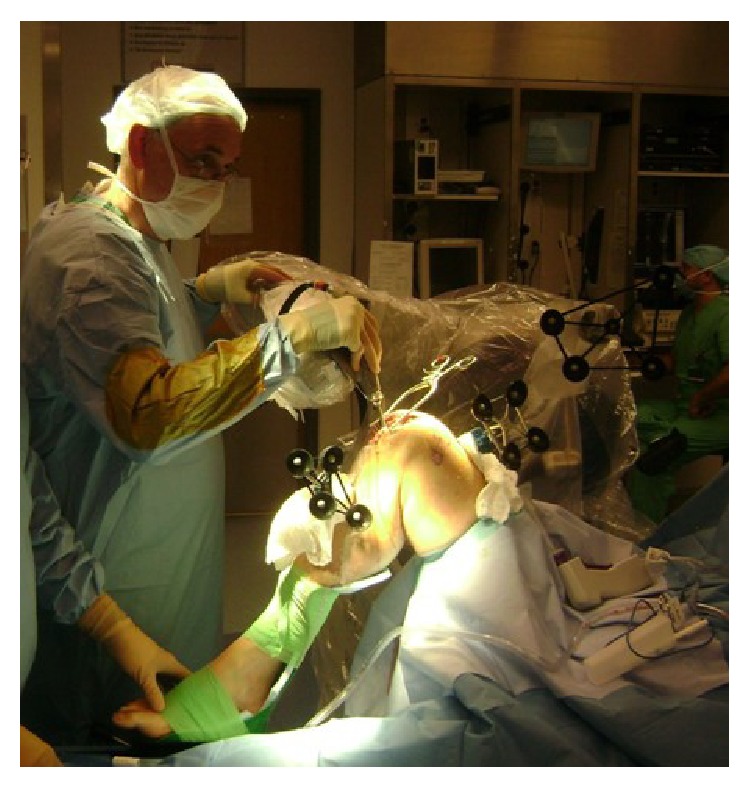
Placement of the femoral and tibial marker array.

In our clinic, we use MAKO Tactile Guidance System. In this system, registration of the patients' anatomy via regular navigation-based reference markers allows for a dynamic tracking of femur and tibia. The robot movements are independent of the patient's positioning or movement, which eliminates the necessity for further rigid fixation device and thus reduces potential complications such as infection, iatrogenic fractures, and soft tissue injury (Figure 3).

Another advantage of the MAKO system is the burring mechanism that permits the creation of individual bony surfaces of any shape with greater precision compared with regular UKA and BiKA cutting guides. This is important medially but even more important laterally because the patella is in the way of conventional cutting guides where the robot only needs space for the 4 mm burr. As demonstrated by Plaskos et al., usage of conventional instruments in bone cuts resulted in errors ranging from 0.6° to 1.1° in varus-valgus and 1.8° in flexion-extension, thus diminishing the accuracy of knee arthroplasty [47]. In the Mako procedure a press-fit cavity for the implant is created preserving the remaining bone surface, which can be very useful for revisions and conversions to total knee prosthesis [48].

### 5.1. Limitations

The main limitation of our study is that it represents retrospective study where patients were not randomized and not compared to a control group. The study contains relatively small sample size with only short-term and mid-term followup. Furthermore, the radiographic followups are done on short-film weight-bearing radiographs, without considering the mechanical axis on plain, long-film weight-bearing radiographs. The functional outcome is only assessed with Oxford Knee Score.

## 6. Conclusion

Robotic assisted bicompartmental arthroplasty using broad indications and only excluding patients with severe deformity and those that have less than 4 mm of joint space in the surviving compartment demonstrated 83% good to excellent results as measured by the Oxford score in 30 knees followed for an average (27) months (12–54).
